# Supplementary material for: Parents’ online coaching in the early intervention home program for toddlers with autism spectrum disorder during the COVID-19 pandemic: Manual development and feasibility study
Source: PLoS One. 2024 Oct 15;19(10):e0309407. doi: 10.1371/journal.pone.0309407 (PMC11478828; doi:10.1371/journal.pone.0309407)
Supplement: Attachment — Submitted filename: Response to Reviewers.docx [file pone.0309407.s002.docx]

**Manuscript ID:** [PONE-D-23-43595R1] - [EMID:6140440efd5fb4d1]

**Tittle:** Parents’ Online Coaching in the Early Intervention Home Program for Toddlers with Autism Spectrum Disorder During the COVID-19 Pandemic: Manual Development and Feasibility Study

| Reviewer #1: | |
| --- | --- |
| - The authors have to clearly mention in the abstract and the objective that this study is just a feasibility study done with 8 parents/participants, which is still missing. This would help a reader to be aware of it right away when they think about the findings of this study, or other researchers would get a hint that they want to validate this scale on a different population before using it. | Thank you for the comment. We have added ‘feasibility’ and (n=?) for each of the stages on p.2 in the abstract section. |
| Reviewer #3 | |
| - The evaluation of the feasibility of the manual was conducted with a sample size of only 8 parents, which is insufficient to draw meaningful conclusions. | Thank you for the comment. Yes, we do agree that 8 participants are insufficient to draw a meaningful conclusion. However, this study is a feasibility study to test the manual development of a specific population before an intervention study can be carried out. Any suggestions and comments from the feasibility study will be improved and implemented for the intervention study phase. We have added more information on the feasibility study on p.9 – Line 180-183. Data saturation on p.22. |
| - The absence of a control group limits the ability to evaluate the effectiveness of your manual in comparison to conventional parenting methods. | Thank you for the comment. We understand your concern. However, this feasibility study focuses solely on validating the feasibility of the manual and the research design. We have added further explanation on p.19. |
| - The qualitative nature of your study makes it challenging to derive robust and generalizable findings. | Thank you for the suggestion and concern raised. However, we designed this as a feasibility study to explore the acceptance of parents using this manual first before a larger RCT study is to be conducted, therefore no control group was planned at this phase. However, future studies on a larger scale will involve a control group. We added this on p. 22. |
| - Suggest conducting a second phase of research with a larger sample size and incorporating more quantitative measures. This would enhance the rigor of your study and provide stronger evidence for the feasibility and effectiveness of your manual for ASD caregivers. | Thank you for the suggestion. Further studies are required to observe the effectiveness and use of the manual with a larger population. We added this on p. 22. |
