# Supplementary material for: Parents’ online coaching in the early intervention home program for toddlers with autism spectrum disorder during the COVID-19 pandemic: Manual development and feasibility study
Source: PLoS One. 2024 Oct 15;19(10):e0309407. doi: 10.1371/journal.pone.0309407 (PMC11478828; doi:10.1371/journal.pone.0309407)
Supplement: Attachment — Submitted filename: Plos One Respond to Reviewer comments.pdf [file pone.0309407.s001.pdf]

Manuscript ID: PONE-D-23-43595

Tittle: Parents’ Online Coaching in the Early Intervention Home Program for Toddlers with Autism Spectrum Disorder During the COVID-19 Pandemic: Manual evelopment and Feasibility Study

Reviewer #1:	
• one in 150 children, and 1 in 54 - Are there across the world or US. Please mention it explicitly where this prevalence rate are from. And cite the right references.	Thank you for the suggestion. We have amended the prevalence rate in the introduction section, p.1.
• Page 4 - line 71: corelated - correlated	Thank you for the suggestion. We have corrected the word- correlated in p.4 – line 73.
• getting a diagnosis is reported as 21.5 - What is 21.5 here? Is that age, months, years?	Thank you for the suggestion. We have added this on p.4 – line 85.
• But one of my main concern is the validity of this manual. The feasibility study was conducted only 8 participants putting this work at borderline. If this work has to reach out to people to really change the lives of many toddler with ASD, the current validation with 8 parents/participants is not enough. I may suggest the authors to go for a short paper rather than a full paper. If this work is about to be published, then I would suggest the authors to mention in the number of participant in the abstract and also clearly state that this is just a preliminary study and this manual will not be supplied until they conduct a large study with handful of participants.	Thank you for the suggestion. We have added this on p.2 – Line 37-38 and p. 22 line 399.
Reviewer #2	
• There is a need for a control group with no parent coaching that will validate the benefits of the intervention program developed in this study.	Thank you for the suggestion. We did this as a feasibility study to explore the acceptance of parents using this manual, no control group was planned for this phase. However, future studies on a larger scale will involve a control group.

• 2. Are there any other home-based intervention programs that can be compared with the results of this study?	Thank you for the suggestion. We compare the other similar program results on p.20-21 from references 40.
• 3. Most of the results are qualitative with no statistical analysis. Incorporating quantitative data with statistical analysis will strengthen the results of this study.	Thank you for the suggestion. This study was designed to be qualitative study. We will do the quantitative data with statistical analysis on our next phase of study.
• 4. On page 14, line 276 please expand and clarify “this indicated that the program manual showed excellent content validity”. Most of the readers are not familiar with this value.	Thank you for the suggestion. We have added further explanation in p. 14, line 271-274.
• 5. The limitations of data collection through online platforms especially involving ASD population should be thoroughly discussed.	Thank you for the suggestion. We have added in p. 21.
• 6. There is a need to update the references with the relevant citations from the year 2023 and 2024.	Thank you for the suggestion. We have added in relevant 2023 and 2024 references as suggested.
